# Screening and Identification of Reference Genes Under Different Conditions and Growth Stages of *Lyophyllum decastes*

**DOI:** 10.3390/ijms262211004

**Published:** 2025-11-13

**Authors:** Yun-Qi Hui, Huan-Ling Yang, Yu-Qing Zhang, Chen-Zhao Zhu, Li-Ping Xi, Chun-Yan Song, Zheng-Peng Li, E-Xian Li, Shu-Hong Li, Yong-Nan Liu, Rui-Heng Yang

**Affiliations:** 1 Hunan Provincial Key Laboratory of Forestry Biotechnology, Central South University of Forestry & Technology, Changsha 410004, China; yqhui115@163.com (Y.-Q.H.); 18390553661@163.com (Y.-Q.Z.); 2 Institute of Edible Fungi, Shanghai Academy of Agricultural Sciences, Shanghai 201403, China; yanghuanling@saas.sh.cn (H.-L.Y.); zhuchenzhao0811@163.com (C.-Z.Z.); xiliping@saas.sh.cn (L.-P.X.); s62209760@163.com (C.-Y.S.); lizp_ln@126.com (Z.-P.L.); 3 State Key Laboratory for Conservation and Utilization of Bio-Resources in Yunnan, Key Laboratory for Southwest Microbial Diversity of the Ministry of Education, Yunnan University, Kunming 650032, China; xiaogaogao4850@126.com (E.-X.L.); shuhongfungi@126.com (S.-H.L.)

**Keywords:** *Lyophyllum decastes*, real-time fluorescence quantitative PCR, internal reference gene, culture conditions

## Abstract

Internal reference genes are a prerequisite for ensuring the accuracy of gene verification experiments, but few relevant studies on *Lyophyllum decastes* have investigated the growth cycle and different environmental conditions. In this study, the qPCR results of 22 house-keeping genes were analyzed using *GeNorm*, *BestKeeper*, *NormFinder* and *RefFinder*. The results revealed that the most stable gene differed under different conditions. Across all developmental stages and under hot, cold, acidic, alkaline, and salt conditions, *UBCE* gene displays the greatest expression stability. However, *EF1b*, *β-ACT*, *HSD17B3*, and *Cyb* presented the greatest stability under cold, heat, and acidic conditions, and heavy metal exposure, respectively. To screen for genes suitable for all conditions, *RefFinder*’s ranking results revealed that *UBCE* and *EF1b* ranked in the top 2, demonstrating the highest gene expression stability. In contrast, *Cyb* was positioned at the bottom of the comprehensive ranking table. This study not only revealed potential factors affecting the suitability of reference genes but also identified optimal reference genes from a set of candidate genes across diverse conditions.

## 1. Introduction

*Lyophyllum decastes* (Fr.) Singer belongs to the Tricholomataceae and is a low-temperature type of edible mushroom. Owing to its abundant polysaccharide, protein, cellulose, and vitamin contents, it is widely recognized for its prominent bioactive properties, including antitumor, immunoregulatory, antioxidant, antidiabetic, and hypolipidemic effects [1,2,3,4]. The growing demand for this mushroom, coupled with its potential for large-scale commercial cultivation, highlights its significant economic value and reinforces its role in both the food industry and broader market [5]. In 2023, China produced 442,200 tonnes of *L*. *decastes*, accounting for the largest global share and reinforcing its position as the world’s leading producer. However, several critical genetic questions, including the developmental mechanisms of the fruiting body and the functions of some key genes, remain unanswered and are closely related to improvements in the cultivation of this fungus. Accurately determining gene expression is highly important for solving these problems, as it provides fundamental insights into how these processes are regulated at the molecular level [6].

Since the genome sequencing of *L. decastes* was completed, research on the functional expression of *L. decastes* has gradually increased [7,8,9,10]. qRT–PCR has become one of the most commonly used and effective methods for comparing the differences in gene expression among experimental samples [11]. The stability of reference genes, which are used as standard indicators in qRT–PCR experiments, determines the accuracy of the detection results [12,13]. Housekeeping genes are often selected as reference genes and standardized to reduce the error range effectively in research [14]. However, in recent years, studies have shown that the expression levels of reference genes used in other species do not remain constant under different experimental conditions [15]. Therefore, it is crucial to assess the stability of reference genes under specific experimental conditions prior to performing quantitative experiments [16]. There are many published articles on the selection of appropriate reference genes for different mushrooms, including *Agaricus bisporus* [17], *Armillaria mellea* [18], *Auricularia cornea* [19], *Ganoderma lucidum* [20], *Pleurotus ostreatus* [21], *Ophiocordyceps sinensis* [22], *Wolfiporia cocos* [23], *Auricularia heimuer* [24], *Flammulina filiformis* [25], *Inonotus obliquus* [26], *Lentinula edodes* [27], *Tricholoma giganteum* [28], *Morchella importuna* [29], *Phlebopus portentosus* [30], *Pleurotus eryngii* [31], *Tuber melanosporum* [32] and *Volvariella volvacea* [33]. These results revealed that different reference genes are required for different species and under different conditions.

Currently, relatively few reports exist on the reference genes of *L. decastes*, which cannot meet the needs of in-depth research on gene expression and its regulatory functions. Liang et al. completed the selection of reference genes for different developmental stages and tissues in *L. decastes* [34]. However, previous research revealed that the expression levels of different genes vary significantly not only at different developmental stages but also in different parts of fruiting bodies and are even more related to different culture environments [30,33]. In *V. volvacea* and *P. portentosus*, stresses such as acid, alkaline, temperature, and oxidation are considered [30,33]. The selection of well-validated reference genes provides a parsimonious and functionally sufficient strategy for normalization when their expression stability has been confirmed under the specific experimental conditions [35]. The reference genes of *L. decastes* under different conditions need to be further studied [36].

Our study aimed to screen reference genes that are stably expressed under different conditions, including acid, alkaline, salt, cold, heat, and heavy metal conditions, providing a foundation for subsequent investigations into the genetic background of *L. decastes*.

## 2. Results

### 2.1. Total RNA Quality Assessment

**Figure 1:**
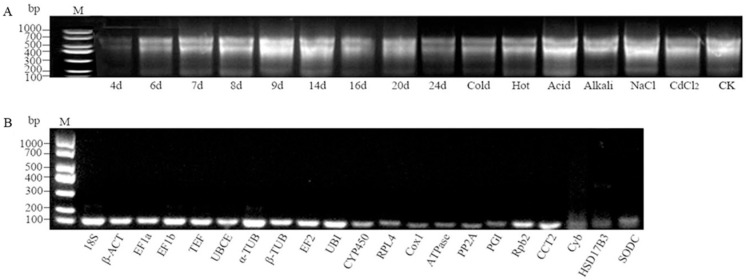
Agarose gel electrophoresis (1%) of the PCR amplicons and RNA extraction (10 μL). (**A**) RNA extraction; (**B**) PCR amplicons of cDNA.

The OD260/OD280 ratios of all the extracted RNA samples ranged from 1.96 to 2.15 (Table S1). The results of integrity testing using 1.0% agarose gel electrophoresis are shown in Figure 1A. Clear 28S and 18S subunit bands and no obvious degradation were observed, indicating that the fragments were intact and good quality. This suggested that this material was suitable for subsequent experiments. To confirm the specificity of the primers with Tm 60 °C, agarose gel electrophoresis was conducted. High specificity was determined by the presence of a single band of the expected size and the absence of primer dimers from each primer pair in the cDNA samples (Figure 1B). Therefore, Tm 60 °C was subsequently used as the annealing temperature for qPCR.

### 2.2. Analysis of Ct Values of Candidate Reference Genes

**Figure 2:**
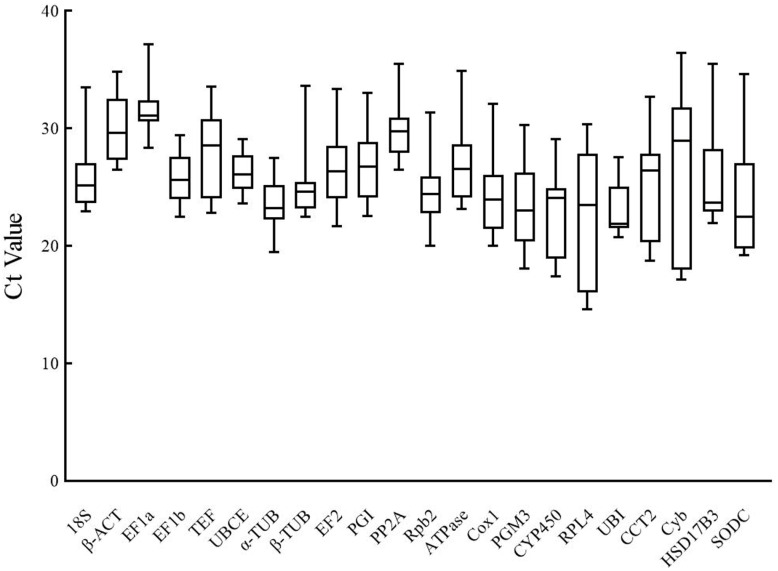
*Ct* values of candidate reference genes.

The lower *Ct* value in the qRT–PCR results revealed a greater gene expression level. The analysis indicated that the *Ct* values ranged, for each gene under different growth stages and conditions, from 14.5 (salt, *RPL4*) to 37.1 (alkaline, *EF1A*) (Figure 2). *EF1A* presented the smallest range of *Ct* values, differing by 3.07 cycles, whereas *Cyb* presented the greatest variation, differing by 13.94 cycles (Figure 2).

### 2.3. Analysis of the Expression Stability of Candidate Genes

#### 2.3.1. *geNorm* Analysis

*geNorm* is a widely accepted program used for screening reference genes by qRT–PCR and determining the optimal number of reference genes among tested candidate genes. It achieves accurate normalization in a given sample panel by calculating the geometric mean of a user-defined number of reference genes. This algorithm identifies reference genes with high stability by calculating the M value for each candidate gene.

**Figure 3:**
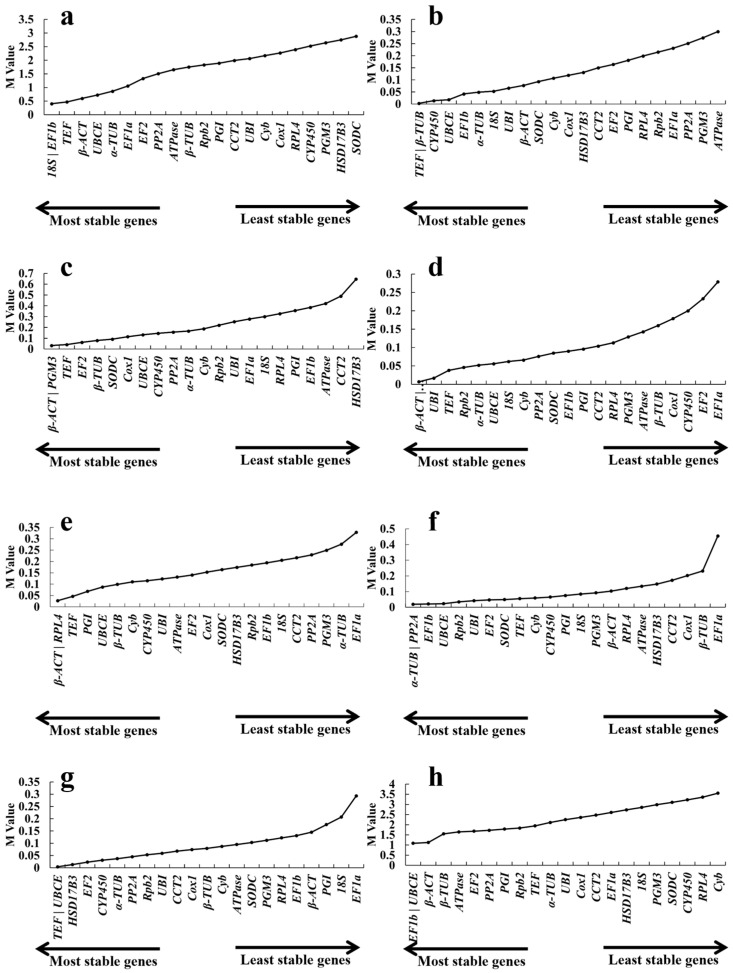
*geNorm* analysis of 22 candidate reference genes under different conditions. Different growth stages of fruiting bodies (**a**), cold (**b**), heat (**c**), pH 4.0 (**d**), pH 9.0 (**e**), 1% NaCl (**f**), 1% CdCl_2_ (**g**), and total (**h**).

*TEF* and *UBCE* presented the highest stability under heavy metal (CdCl_2_) exposure, with M values of 0.004—which were lower than those of all the other genes analyzed in this study (Figure 3g). In addition, *TEF* was also the most stable gene for qRT–PCR under cold conditions (Figure 3b). *β-ACT* exhibited the highest stability under heat, acid, and alkali conditions, with M values of 0.07, 0.17, and 0.17, respectively. This finding indicated that it was a relatively optimal reference gene for these conditions (Figure 3c–e). *18S* and *EF1b* were the most stable in different developmental stages (Figure 3a). Considering all the different conditions, the combined analysis revealed that *EF1b*, *UBCE*, *β-ACT*, *β-TUB*, and *ATPase* were the universal genes used as internal reference genes (Figure 3h). The M values of *CYP450*, *RPL4* and *Cyb* were the highest, revealing that the expression of these three genes varied greatly (Figure 3h).

**Figure 4:**
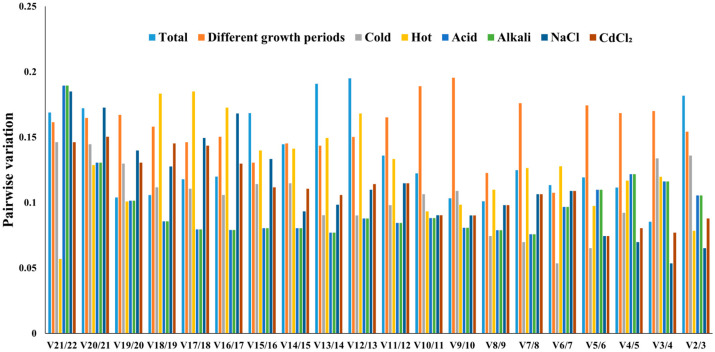
The pairwise differences in Vn and Vn + 1 analyzed using *GeNorm* (Vn and Vn + 1 < 0.150).

*geNorm* can also be employed to determine the optimal number of reference genes for normalization. Specifically, the pairwise variation (Vn/Vn + 1) between the normalization factors is calculated for all samples, and 0.15 is suggested as the threshold value. On this basis, the pairwise variations were calculated and are listed in Figure 4. As shown, the two most stable reference genes were adequate for reliable normalization across all samples except under all conditions. Three genes were required for normalization when V3/4 < 0.09 was used for all the conditions, as the pairwise variation V2/3 value was 0.18.

#### 2.3.2. *NormFinder* Analysis

**Figure 5:**
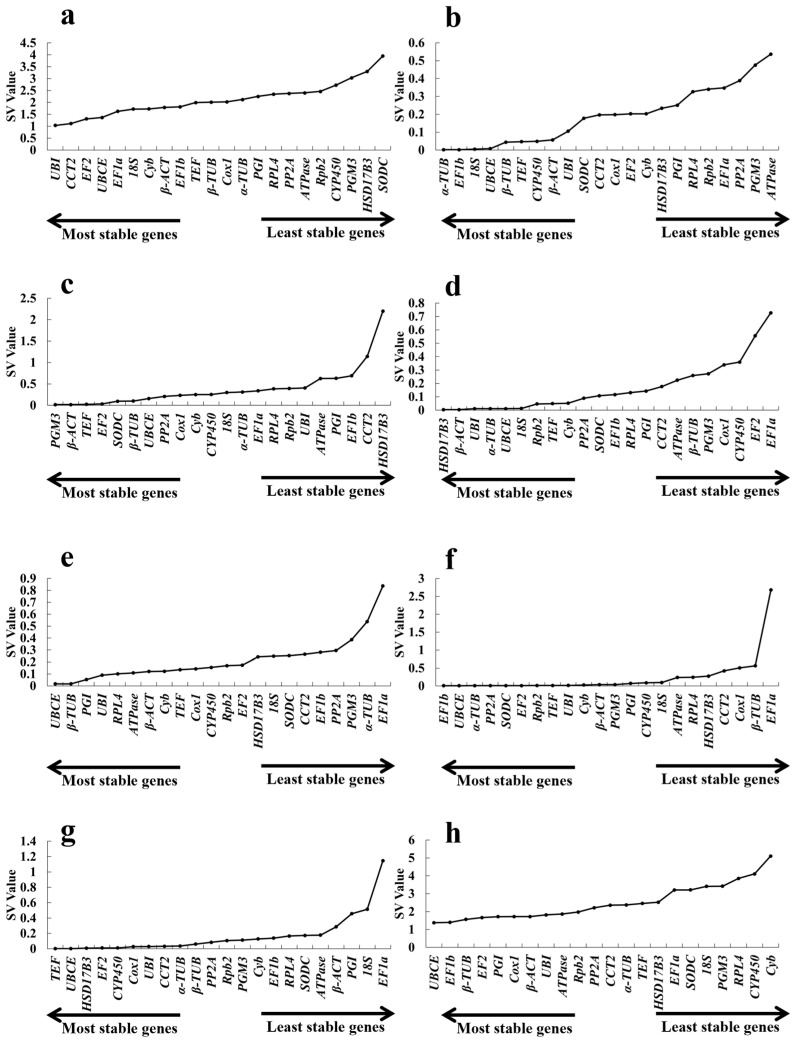
*NormFinder* analysis of 22 candidate reference genes under different conditions. Different growth stages of fruiting bodies (**a**), cold (**b**), heat (**c**), pH 4.0 (**d**), pH 9.0 (**e**), 1% NaCl (**f**), 1% CdCl_2_ (**g**), and total (**h**).

*NormFinder* evaluates the expression stability of each single reference gene and takes into account intra- and intergroup variations for normalization. For each candidate gene, *NormFinder* provides a stability value (*SV)* related to expression variation. *UBI* was the most stable across different developmental stages; however, its *SV* was significantly higher than those of the other treatments. The *SV*s of *α-TUB* and *EF1b*, *TEF* and *UBCE* under cold and CdCl_2_ conditions were 0.002, which were the lowest in this study across all the treatments (Figure 5b,g). *UBCE* was also the most stable gene in alkaline and salt environments (Figure 5e,f). In addition to *UBCE*, the *SVs* of *β-TUB* and *EF1b* were both 0.002 under the same conditions. The *β-ACT* and *HSD17B3* genes presented the most stable potential when treated with acid (Figure 5d). *PGM3* and *β-ACT* presented the highest stability at high temperatures. The values of *UBI* and *CCT2* across all the developmental stages were 1.034 and 1.112, respectively, and these were lower than those of the other genes in the same stage and much higher than those of the other most stable genes in all the treatments used in this study (Figure 5c). Like in the abovementioned analysis, different genes presented different expression stabilities. To obtain a consensus internal reference gene, combination analysis was also used. The results revealed that the *SVs* of *UBCE* and *EF1b* were 1.378 and 1.4, respectively, indicating that they have the potential to adapt to various environments (Figure 5h). *RPL4*, *CYP450* and *Cyb* were the most unstable genes across all the treatments, with *SVs* higher than 4.00 (Figure 5h).

#### 2.3.3. *BestKeeper* Analysis

**Figure 6:**
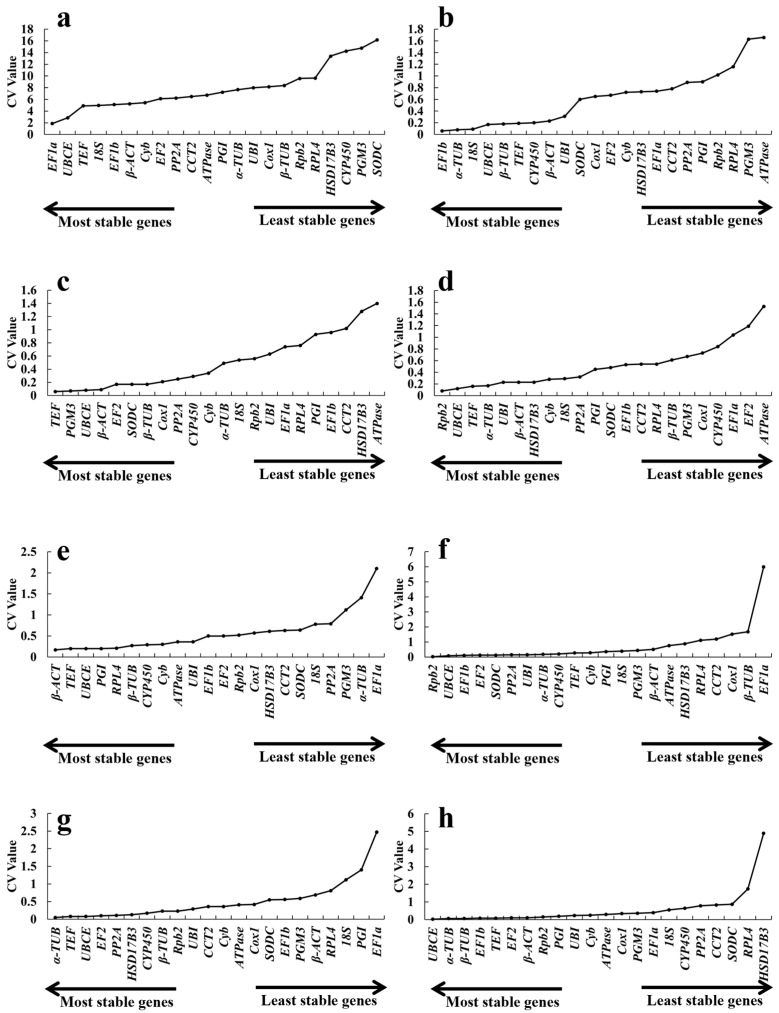
*BestKeeper* analysis of the *CV* values of 22 candidate reference genes at different growth stages: fruiting bodies (**a**), cold (**b**), heat (**c**), pH 4.0 (**d**), pH 9.0 (**e**), 1% NaCl (**f**), 1% CdCl_2_ (**g**), and total (**h**).

*BestKeeper* assesses the expression stability of reference genes by analyzing raw *Ct* data and calculates stability indices on the basis of the standard deviation (*SD*) and coefficient of variation (*CV*) of all candidate genes evaluated [37]. When the CV and SD are smaller, the gene is more stable. According to the suitable reference criterion, genes with SDs > 1.0 are considered unstable and should be avoided. Only the *SD* values of *HSD17B3* and *EF1a* exceeded 1.0 across developmental stages, heat, and salt treatments, with values of 2.57, 1.66, and 2.04, respectively (Figure 6a,c,f). Consequently, both genes were excluded from further analysis. In all other cases, the *SD* values were less than 1.0. Notably, *Rpb2* and *α-TUB* presented the lowest *SD* values (0.01) under the salt and CdCl_2_ treatments (Figure 6f,g). Under the same conditions, *TEF* and *UBCE* were also relatively stable genes with low *SD* values. Among them, *UBCE* was also one of the most stable genes in the cold and alkaline treatments (Figure 6b,e). *β-ACT* was identified as the most suitable reference gene under heat and alkaline conditions (Figure 6c,e). *TEF* also showed the greatest stability in samples subjected to cold, acidic conditions, and CdCl_2_ treatments (Figure 6b,d,g). Furthermore, *18S* was the most stable gene under cold conditions (Figure 6b). A comprehensive evaluation integrating all the treatments was conducted to screen for reference genes with broad applicability. These results indicated that *EF1a* and *UBCE* could be used as universal internal reference genes.

**Figure 7:**
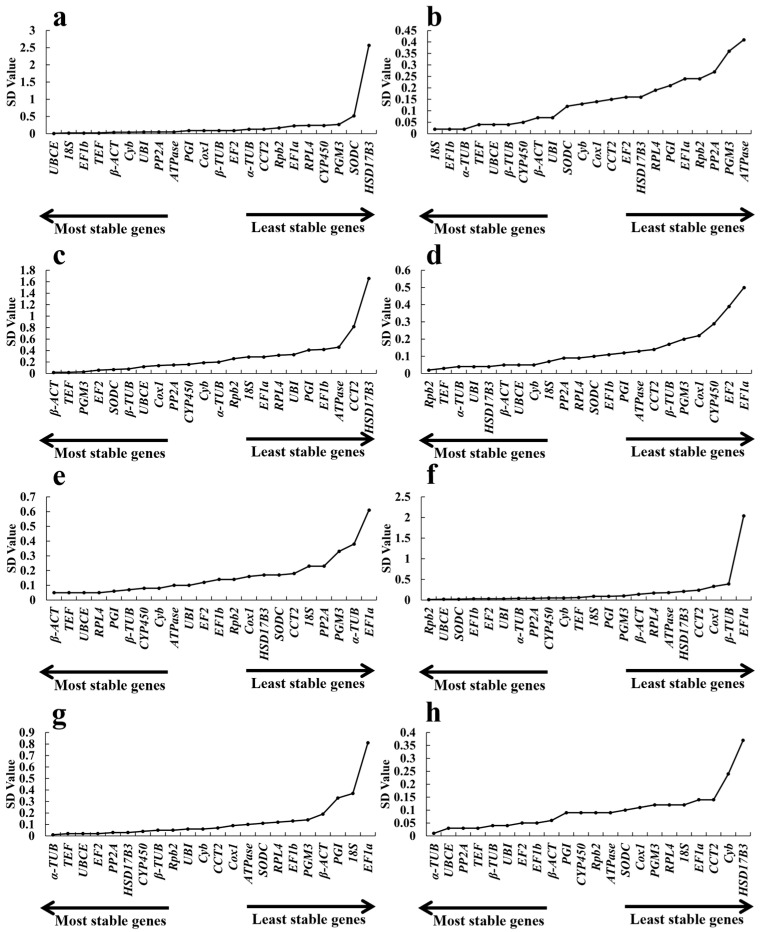
*BestKeeper* analysis of the SDs of 22 candidate reference genes at different growth stages: fruiting bodies (**a**), cold (**b**), heat (**c**), pH 4.0 (**d**), pH 9.0 (**e**), 1% NaCl (**f**), 1% CdCl_2_ (**g**), and total (**h**).

During the developmental stages, *EF1a*, *UBCE*, *TEF*, *18S* and *EF1b* were the five genes with the most stable expression, as revealed by their low *CV* values (Figure 7a). However, *EF1a* had the least stability under alkaline, salt and CdCl_2_ conditions. *β-ACT* (0.17), *Rpb2* (0.02) and *α-TUB* were stable genes (Figure 7e,f,g). The genes with the lowest values under the other treatments (cold, heat and acid, respectively) were *EF1b* (followed by *α*-*TUB*, *18S*, *UBCE* and *β*-*TUB*), *TEF* (followed by *β-ACT*, *PGM3*, *EF2* and *SODC*) and *Rpb2* (followed by *TEF*, *α*-*TUB*, *β*-*ACT* and *UBI*) (Figure 7b–d). Although different genes had different stabilities under different conditions, *UBCE* in all the treatments ranked among the genes with the highest stable expression, followed by *α-TUB* (0.06), *β-TUB* (0.06), and *EF1b* (0.08). The Δ*C* value of *HSD17B3* was 4.89, the largest value in this study, indicating the worst expression stability (Figure 7h).

#### 2.3.4. Comprehensive Stability Analysis of the Reference Genes

*RefFinder* integrates the results from the widely used algorithms *geNorm*, *Normfinder* and *BestKeeper* to make comparisons and rank the tested candidate reference genes [38]. Using the rankings from each algorithm, we assigned weights to individual genes proportional to their stability scores and computed the geometric mean of these weights to establish the final comprehensive ranking. On the basis of the comprehensive ranking, *UBCE* emerged as the most suitable reference gene for alkaline and salt conditions, as well as across developmental stages. *EF1b*, *TEF*, *α-TUB*, and *TEF* ranked highest in the analyses for the cold, heat, acid, and CdCl_2_ treatments, respectively. When all the treatments were combined, *UBCE*, *EF1b*, *β-TUB*, *β-ACT* and *EF2* ranked in the top five positions and presented the greatest gene expression stability (Table 1). *PGM3*, *SODC*, *CYP450*, *RPL4* and *Cyb* are located at the bottom of the comprehensive table.

### 2.4. Verification of Reference Gene Stability

**Figure 8:**
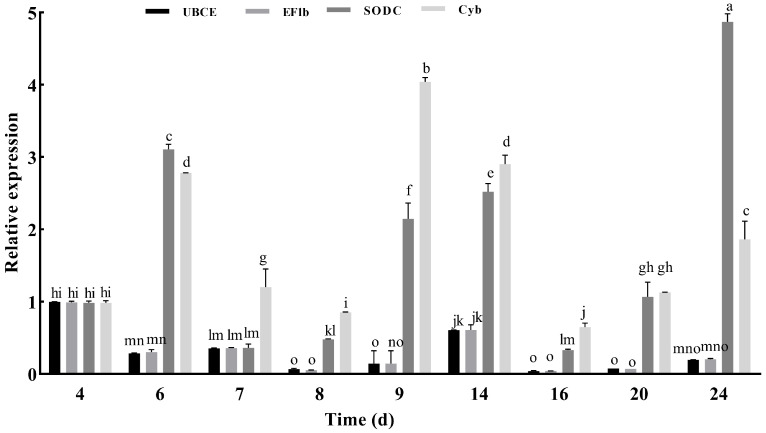
Relative expression of *UBCE*, *EF1b*, *Cyb* and *SODC* using *lac 2* as reference. Different lowercase letters indicate significant differences (*n* = 3, *p* < 0.05).

To further verify the stability of the selected reference genes, the relative expression of the key gene *lac2* in response to fruiting body developmental stages was monitored. During the entire growth period of *L. decastes*, the expression levels of *UBCE*, *EF1b*, *Cyb*, and *SODC* were determined using *lac2* as a reference gene (Figure 8). Normalization results with *UBCE* and *EF1b* showed similar expression patterns, with the highest levels observed at 4 d. In contrast, *Cyb* and SODC exhibited distinct trends: *SODC* reached its peak at 24 d, while the highest expression of *Cyb* occurred at 9 d. Comprehensive analysis indicated that throughout all growth stages, *UBCE* and *EF1b* were the most stable candidate reference genes, whereas *Cyb* and *SODC* were the least stable.

## 3. Discussion

In this study, the expression stability of 22 candidate reference genes was evaluated and ranked. This was the first in-depth study that examined the stability of several genes serving as internal controls in RT–qPCR studies. Different conditions were considered instead of only the growth and development periods in *L. decastes.* The results revealed that *UBCE* was the most suitable reference gene under different conditions and different developmental stages. These findings could serve as a reference for gene expression analysis in this fungus. Moreover, these findings highlight the importance of selecting appropriate reference genes.

In this study, the most authoritative algorithms or methods (*geNorm*, *BestKeeper*, *NormFinder*, and *RefFinder*) were utilized to screen for the most appropriate reference genes. These algorithms are frequently applied in the fields of plants, animals, and fungi [39]. In edible fungi, the best reference genes (control genes) have been identified using these tools and include [17,18,24] *C. militaris* [40], *F. filiformis* [25], *G. lucidum* [20], *Hymenopellis radicata* [41], *I. obliquus* [26], *L. sordida* [42], *T. giganteum* [28], *M. importuna* [29], *O. sinensis* [22], *P. eryngii* [31], *P. ostreatus* [21], *T. melanosporum* [21], *V. volvacea* [33] and *W. cocos* [23]. However, the results from the different methods are not always consistent [30,33]. When the stability of the reference genes in this study was analyzedc *geNorm* and *NormFinder* often reached the same conclusion [30,31]. According to these criteria, *BestKeeper* selected the most stable reference gene, *EF1A*, whereas *UBCE and EF1b* were identified as the most stable genes in the other two software programs. The analysis results of the BestKeeper software showed certain discrepancies compared with those of these two software programs [31,36,43,44]. This may be because *BestKeeper* relies on the standard deviation of candidate reference genes to evaluate their stability, whereas *geNorm* and *NormFinder* assess reference genes according to the cumulative standard deviation and pairwise stability of the genes [33]. Therefore, it is necessary to analyze the RT–qPCR results using multiple analysis software programs, use *RefFinder* software to rank the analysis results comprehensively, and finally screen the optimal reference genes [36]. Although the integration results from *RefFinder* may be influenced by the distinct statistical assumptions underlying the included algorithms, they could offset the potential integration bias of a single tool and enhance the precision of gene set selection [35]. In this study, the reference genes *UBCE* ultimately selected demonstrated high stability and ranked on the top in the vast majority of independent algorithms.

The first study that reported an investigation of suitable internal control genes was conducted by Liang et al., who evaluated 10 candidate genes across different developmental stages and various parts of fruiting bodies in *L. decastes* [34]. However, in studies on fungal genetics, response mechanisms to various environmental conditions have been identified in many instances [30,33]. This underscores the need to have a reference gene with a wider scope of application. Therefore, in studies of *V. volvacea* and *P. portentosus*, different factors were considered [18,30]. This was aimed mainly at obtaining a reference gene that exhibited stable expression across all environments. This is the first study to screen stable internal genes for acid, alkali, temperature and heavy metal contents in *L. decastes*. In addition, a total of 22 candidate reference genes, including the 10 genes in the previously published related study, were utilized in this study [34], and these genes were commonly tested in other studies on mushrooms. The most suitable internal genes often differ in different species and under different conditions [30,33]. Almost all related studies reported this phenomenon. In this study, ubiquitin-conjugating enzyme (*UBCE*) emerged as the most suitable internal reference gene across a wider range of environmental conditions. In addition, *UBCE* was also reported to be the ideal reference gene for *Oreochromis niloticus* [45], *Oocystis borgei* [46], and *Piper* species [47]. Genetic investigations have uncovered that *UBCE* orchestrate an impressively wide array of functions, including DNA repair, sporulation, cell cycle progression, peroxisome biogenesis, membrane—protein degradation, heat shock resistance, and cadmium tolerance in plants and animals [48]. In mushrooms, *UBCE* may be indispensable for growth, development, and response to stresses [49,50]. In *V. volvacea*, *UBCE* was correlated with the cryogenic autolysis [49]. However, the functions of these related enzymes in mushrooms have not been uncovered now. In *L. decastes, CYP450*, *RPL4* and *Cyb* were the most unstable genes. This result was in agreement with the evaluation of *CYP450* in *Cicer* species [51]. These observations were intriguing since they contrast with studies in *Poria cocos* and *G. lucidum*, where the *CYP* and *RPL4* genes were expressed most stably [20,47]. This inconsistency might be caused by species differences. Another study on selecting internal reference genes for *L. decastes* reported that, based on *RefFinder* results, *HSD17B3* exhibited the most stable expression across developmental stages [34]. However, in our study, *HSD17B3* showed the opposite trend in expression stability. In our stability ranking, *UBCE* and *18S* performed significantly better than *HSD17B3*, indicating that *HSD17B3* was less stable as a reference gene compared to these two genes. Notably, *18S* has also been identified as the optimal reference gene for different developmental stages in *L. edodes* [27] and *T. melanosporum* [32].

In addition to the in silico analyses, *Lac2* (a laccase) was used to validate the stability of certain genes across developmental stages. This same verification strategy has been employed in other studies [52,53,54]. Laccases are known to participate not only in lignin degradation but also in regulating fruiting body morphogenesis and pigment formation in mushrooms [54,55,56,57,58,59,60]. Normalization results using *UBCE* and *EF1b* showed consistent expression patterns, with no significant differences detected across all stages. In contrast, *Cyb* and *SODC* exhibited distinct expression trends. These findings indicated that UBCE and EF1b are more stably expressed than *Cyb* and *SODC*, which was consistent with the results described above.

## 4. Materials and Methods

### 4.1. Sample Preparation

The tested strain of *L. decastes* is currently preserved in the National Engineering Research Center for Edible Fungi, Shanghai Academy of Agricultural Sciences. This fungus was cultivated to promote the growth of mycelia on potato dextrose agar (PDA) at 25 °C in the dark as previously described [34]. The growing mycelia were treated with NaCl (1%), CdCl_2_ (1%), HCl (pH 4.0), and NaOH (pH 9.0) at the optimal growth temperature of 25 °C 160 rpm/min in 150 mL potato dextrose broth (PDB) for 7 days [30,33]. For the temperature experiments, at the optimal growth temperature of 25 °C, the mycelia grown in 150 mL of PDA were incubated for 6 days and then treated at 0 °C and 35 °C for 24 h in the dark. The fruiting bodies were collected at culture times of 4 d, 6 d, 7 d, 8 d, 9 d, 14 d, 16 d, 20 d and 24 d. All the samples were chopped into small pieces (2 mm), and immediately frozen in liquid nitrogen and stored at −80 °C before RNA extraction. Three independent biological replicates were tested for each treatment.

### 4.2. Total RNA Extraction and cDNA Synthesis

The total RNA of fruiting bodies was extracted using a Redzol reagent kit from Shanghai Qingke Biotechnology Co., Ltd. (Shanghai, China). RNA integrity was detected using 1% agarose gel electrophoresis at 120 V for 10 min. RNA concentration and purity were measured using a nucleic acid–protein analyzer. The reverse transcription reaction system was prepared in a total volume of 20 μL, containing 5 μL of 4× One-Tube RT SuperMix, 1 μg of RNA template (with equal amounts of RNA used for different samples [17,18,19,20,21,22,23,24,25,26,27,28,29,30,31,32,33,34,35,36]), and double-distilled water to bring the volume to 20 μL. Reverse-transcribed cDNA was synthesized using the PrimeScript^TM^ RT Reagent Kit for qRT–PCR according to the manufacturer’s instructions. All the samples were stored at −80 °C for later use.

### 4.3. Selection of Reference Candidate Genes and Primer Design

Reference genes were screened in line with the criteria published in the study of screening and validation of reference genes in other species(Table S2). Finally, *18S*, *β-ACT*, *EF1a*, *EF1b*, *TEF*, *UBCE*, *α-TUB*, *β-TUB*, *EF2*, *UBI*, *CYP450*, *RPL4*, *PGM3*, *CCT2*, *Cox1*, *Rbp2*, *Cyb*, *ATPase*, *HSD17B3*, *PGI*, *PP2A* and *SODC* were selected as candidate test reference genes (Table S1). All the sequences were extracted from the genome published previously [8]. Specific primers were designed using the Primer-BLAST online program (https://www.ncbi.nlm.nih.gov/tools/primer-blast/; accessed on 20 April 2024.), with amplicon lengths ranging from 100 to 200 bp. All primers were synthesized by Qingke Biotechnology Co., Ltd. (Shanghai) and stored at 4 °C for later use. The specificity of the amplified products was detected by 1% agarose gel electrophoresis (Table 2).

### 4.4. Fluorescence Quantitative PCR

For real-time fluorescence quantitative PCR, a 2× Universal SYBR qPCR mix (TaKaRa, Kusatsu, Japan) kit was used. The experiment was performed on a Step OnePlus Real-Time PCR instrument(Applied Biosystems, Thermo Fisher Scientific, Shanghai, China). The reaction system consisted of 10 μL of 2× Universal SYBR qPCR mix (blue), 2 μL of template cDNA, 0.4 μL of each primer, and 7.2 μL of RNase-free water. The amplification program was as follows: predenaturation at 95 °C for 1 min; 40 cycles of 95 °C for 10 s and 60 °C for 30 s; and melting curve analysis at 95 °C for 15 min, 60 °C for 30 s (adjust according to the primer *Tm* value), and 95 °C for 15 min. Four technical replicates were set for each reaction.

### 4.5. Data Analysis

After qRT–PCR amplification was completed. The *geNorm* website (https://seqyuan.shinyapps.io/seqyuan_prosper/; accessed on 20 April 2024.), *NormFinder* [61], *BestKeeper* [37], and *RefFinder* [38] were used to analyze the differences in the expression of 22 candidate reference genes.

### 4.6. Experimental Validation

To confirm the validity of the selected reference genes for data normalization, the two most stable and two least stable candidate reference genes were selected [55]. Meanwhile, the expression levels of laccase (*lac2*) across different developmental stages were verified by qRT-PCR. The qRT-PCR amplification conditions were consistent with those described above. For gene expression analysis via qRT-PCR, three technical replicates were included for each biological replicate. In this study, the Duncan multiple range test was used to evaluate the significant intergroup differences in using Analysis of Variance (*ANOVA*).

## 5. Conclusions

In this study, a total of 22 housekeeping genes (*18S*, *β-ACT*, *EF1A*, *EF1B*, *TEF*, *UBCE*, *α-TUB*, *β-TUB*, *EF2*, *UBI*, *CYP450*, *RPL4*, *PGM3*, *CCT2*, *Cox1*, *Cyb*, *ATPase*, *HSD17B3*, *PGI*, *PP2A*, *Rbp2* and *SODC*) were validated under different conditions and at different developmental stages. According to the selection criteria, the reference gene *UBCE* was relatively stable, with broader suitability for different environments. *UBCE* selected in this study not only exhibited excellent average inter-sample stability, but also showed extremely low variability across its three biological replicates at all experimental time points. This indicated that its expression was not affected by minor experimental fluctuations, and thus its stability was highly reliable.

This study provided an important tool for the molecular identification and functional research of germplasm resources of *L. decastes*.

## Figures and Tables

**Table 1:** Comprehensive sequencing of candidate reference genes using *RefFinder*.

Gene Name	Different Growth Periods Ranking	Cold Ranking	Hot Ranking	Acid Ranking	Alkali Ranking	NaCl Ranking	CdCl_2_ Ranking	Total Ranking
*UBCE*	1	2	3	3	1	1	2	1
*EF1b*	3	1	2	8	16	2	4	2
*β-TUB*	12	6	5	5	5	21	3	3
*β-ACT*	9	5	4	4	2	15	7	4
*EF2*	5	15	11	20	11	7	15	5
*PGI*	15	16	8	12	4	12	6	6
*ATPase*	14	20	22	18	8	17	17	7
*PP2A*	13	7	18	19	19	5	5	8
*EF1a*	4	21	19	22	22	22	20	9
*α-TUB*	11	4	12	1	21	3	16	10
*UBI*	6	8	13	6	7	8	8	11
*Rpb2*	17	11	7	16	13	4	12	12
*Cox1*	16	17	16	9	12	20	18	13
*TEF*	8	3	1	2	6	10	1	14
*CCT2*	7	13	10	10	18	19	13	15
*18S*	2	10	9	13	17	13	21	16
*HSD17B3*	21	18	20	15	14	18	22	17
*PGM3*	20	19	17	14	20	14	14	18
*SODC*	22	22	14	11	15	6	19	19
*CYP450*	19	9	6	21	10	11	9	20
*RPL4*	18	12	15	17	3	16	10	21
*Cyb*	10	14	21	7	9	9	11	22

**Table 2:** Characteristics and targets of the qPCR primers.(Note: F represents the upstream primer; R stands for downstream primer.)

Primer Name	Primer sequence (5′-3′)	Length of Product (bp)	*Tm*/°C
*18S*-F	TATTATGGCGACACCGAGGC	191	57.45
*18S*-R	CCCAGCCCAAATGTAACCCT		57.45
*EF1a*-F	CGTGGTAACGTCTGTTCCGA	137	57.45
*EF1a*-R	TGAGCGGTGTGACAATCCAA		59.5
*β-ACT*-F	CTTCCCATTCCCCTGACCTG	126	59.5
*β-ACT*-R	GCGCTTCAAACCCGACTAAG		57.45
*UBCE*-F	GCTAGATCGTTTGTCGCAGC	112	57.45
*UBCE*-R	TGTGACTGCAAGAGTCCGTC		55.40
*TEF*-F	GTCCAGGCCGTTGAACAAAC	107	55.40
*TEF*-R	AAGGGCGAAGATAGCGATGG		57.45
*EF1b*-F	ACCGCTTTTTGCCGAAATCC	185	57.45
*EF1b*-R	TCACCATGAAACTGCCCTCC		55.40
*α-TUB*-F	GACCGAGACCTTATGGAGCG	159	55.40
*α-TUB*-R	GAGGTCTGTGTCGTGTCCTG		57.45
*β-TUB*-F	CGAAAGCTTTAGGAAGTGCCG	99	59.50
*β-TUB*-R	CCGGATGAATGGAAGAGGGG		57.40
*UBI*-F	CTGCGTAACACGGACGAGAT	116	57.45
*UBI*-R	AGCACTTGTGCGATCTGAGG		57.45
*CYP450*-F	ATCCGCTTATCGGACACCTC	124	57.45
*CYP450*-R	GTGGAGCGCATGAATCTCCT		57.45
*RPL4*-F	CATGTTCGCTCCCACCAAGA	156	59.5
*RPL4*-R	AGGGGAACCTCCTCGATCTC		59.5
*EF2*-F	CTGTGCAGAAGAGAACATGCG	103	57.45
*EF2*-R	CTATAGGACTCGGTGGGCAAA		57.45
*PGM3*-F	TCATGATTGCCAGCGAACCT	114	55.40
*PGM3*-R	CGATAATCGGGACCTGGAGC		55.40
*CCT2*-F	GTGAAGCTCGGACACTGTGA	166	57.45
*CCT2*-R	CAGAAAGCGCATCGTGTAGC		57.45
*Cox1*-F	TGGGGTGGTTCTGTCGATTG	181	55.40
*Cox1*-R	CGCGTGGAATGAAAGTAGCG		55.40
*Cyb*-F	GGACCATCCAGACCGTGAAG	170	57.45
*Cyb*-R	GTAGAGGACAACACCGAGGC		59.50
*ATPase*-F	CTGCAGGCCATTTCGTATGC	155	57.40
*ATPase*-R	TCGCTACTCGGATTTCTCGC		57.45
*HSD17B3*-F	TTCCAGCATCGTTGCAGTCT	178	55.40
*HSD17B3*-R	GTTGGCGATAGCAAAGCTCG		55.40
*PGI*-F	TGATCGAGGTCGACTGAGGT	147	57.45
*PGI*-R	GACCATGACCGCACTCTTCA		57.45
*PP2A*-F	TGCGATAGCCATTGTGGGTT	148	55.40
*PP2A*-R	GAGACGATGGCACGAGTAGG		55.40
*Rpb2*-F	GAAGGCGTACTTCGTCCACA	106	57.45
*Rpb2*-R	CACTCTGGAGATCCCTTGGC		59.50
*SODC*-F	CATGACCGAAACATCGACGC	113	57.40
*SODC*-R	ACAATTCGCAACCCATTGCC		57.45

## Data Availability

The original contributions presented in this study are included in the article/Supplementary Materials. Further inquiries can be directed to the corresponding author.

## Supplementary Materials

The following supporting information can be downloaded at https://www.mdpi.com/article/10.3390/ijms262211004/s1.
