# Supplementary material for: Screening and Identification of Reference Genes Under Different Conditions and Growth Stages of Lyophyllum decastes
Source: Int J Mol Sci. 2025 Nov 13;26(22):11004. doi: 10.3390/ijms262211004 (PMC12652064; doi:10.3390/ijms262211004)
Supplement: Supplementary file 1 [file ijms-26-11004-s001.zip › ijms-3951623-supplementary.pdf]

Table S1 The information of RNA and cDNA used for real-time fluorescence quantitative PCR

Samples	OD260/OD280 of RNA	Concentration of cDNA (ng/μL)	Volume (μL)
4d	2.00	675.3	1.48
6d	1.96	1036.5	0.96
7d	2.09	1333.8	0.75
8d	1.99	1659.3	0.60
9d	2.15	1096.8	0.91
14d	2.05	1780.2	0.56
16d	2.00	2030.9	0.49
20d	2.00	2505.7	0.40
24d	1.96	2446.7	0.41
Cold	2.07	1010.2	0.99
Hot	2.00	1551.9	0.64
Acid	2.05	2067.1	0.48
Alkali	1.99	1906.8	0.52
NaCl	2.11	1263.1	0.79
CdCl2	2.11	1043.1	0.96
CK	2.07	1367.7	0.73

Table S2 The information of genes used in this study

Genes	Full name
18S	18S Ribosomal RNA
β-ACT	Beta-actin
EF1a	Eukaryotic translation elongation factor 1 alpha
EF1b	Elongation Factor 1 Beta
TEF	Transcription elongation factor
UBCE	Ubiquitin-Conjugating Enzyme E2
α-TUB	Alpha Tubulin
β-TUB	Beta-tubulin
EF2	Eukaryotic Translation Elongation Factor 2
UBI	Ubiquitin
CYP450	Cytochrome P450
RPL4	Ribosomal protein L4
PGM3	Phosphoglucomutase 3
Cox1	Cytochrome C oxidase subunit 1
ATPase	ATPase
PGI	Glucose-6-phosphate isomerase
PP2A	Protein phosphatase 2A
Rpb2	DNA-directed RNA polymerase subunit 2

CCT2	Chaperonin containing TCP1, Subunit 2
Cyb	Cytochrome b560 subunit
HSD17B3	17 Beta-hydroxysteroid dehydrogenase type 3
SODC	Copper/zinc superoxide dismutase
